# Design and Implementation of the Pre-Clinical DICOM Standard in Multi-Cohort Murine Studies

**DOI:** 10.3390/tomography7010001

**Published:** 2021-02-05

**Authors:** Joseph D. Kalen, David A. Clunie, Yanling Liu, James L. Tatum, Paula M. Jacobs, Justin Kirby, John B. Freymann, Ulrike Wagner, Kirk E. Smith, Christian Suloway, James H. Doroshow

**Affiliations:** 1 Small Animal Imaging Program, Laboratory Animal Sciences Program, Frederick National Laboratory for Cancer Research, Frederick, MD 21702, USA; 2 PixelMed Publishing, Bangor, PA 18013, USA; dclunie@dclunie.com; 3 Image and Visualization Group, Advanced Biomedical and Computational Sciences, Biomedical Informatics and Data Science, Frederick National Laboratory for Cancer Research, Frederick, MD 21702, USA; liuy5@mail.nih.gov (Y.L.); csuloway@gmail.com (C.S.); 4 Cancer Imaging Program, Division of Cancer Treatment and Diagnosis, National Cancer Institute, National Institute of Health, Rockville, MD 20892, USA; tatumj@mail.nih.gov (J.L.T.); jacobsp@mail.nih.gov (P.M.J.); 5 Cancer Imaging Informatics Lab, Frederick National Laboratory for Cancer Research, Frederick, MD 21702, USA; kirbyju@mail.nih.gov (J.K.); freymannj@mail.nih.gov (J.B.F.); 6 Biomedical Informatics and Data Science, Frederick National Laboratory for Cancer Research, Frederick, MD 21702, USA; ulrike@mail.nih.gov; 7 Department of Biomedical Informatics, University of Arkansas for Medical Sciences, Little Rock, AR 72205, USA; KESmith2@uams.edu; 8 Division of Cancer Treatment and Diagnosis, Center for Cancer Research, National Cancer Institute, National Institute of Health, Rockville, MD 20892, USA; doroshoj@mail.nih.gov

**Keywords:** DICOM, pre-clinical, co-clinical, in vivo imaging, animal model, patient-derived xenograft (PDX)

## Abstract

The small animal imaging Digital Imaging and Communications in Medicine (DICOM) acquisition context structured report (SR) was developed to incorporate pre-clinical data in an established DICOM format for rapid queries and comparison of clinical and non-clinical datasets. Established terminologies (i.e., anesthesia, mouse model nomenclature, veterinary definitions, NCI Metathesaurus) were utilized to assist in defining terms implemented in pre-clinical imaging and new codes were added to integrate the specific small animal procedures and handling processes, such as housing, biosafety level, and pre-imaging rodent preparation. In addition to the standard DICOM fields, the small animal SR includes fields specific to small animal imaging such as tumor graft (i.e., melanoma), tissue of origin, mouse strain, and exogenous material, including the date and site of injection. Additionally, the mapping and harmonization developed by the Mouse-Human Anatomy Project were implemented to assist co-clinical research by providing cross-reference human-to-mouse anatomies. Furthermore, since small animal imaging performs multi-mouse imaging for high throughput, and queries for co-clinical research requires a one-to-one relation, an imaging splitting routine was developed, new Unique Identifiers (UID’s) were created, and the original patient name and ID were saved for reference to the original dataset. We report the implementation of the small animal SR using MRI datasets (as an example) of patient-derived xenograft mouse models and uploaded to The Cancer Imaging Archive (TCIA) for public dissemination, and also implemented this on PET/CT datasets. The small animal SR enhancement provides researchers the ability to query any DICOM modality pre-clinical and clinical datasets using standard vocabularies and enhances co-clinical studies.

## 1. Introduction

The use of the Digital Imaging and Communications in Medicine (DICOM) standard for storage and interchange of radiology images is ubiquitous in human clinical imaging environments. DICOM started when the American College of Radiology (ACR) and the National Electrical Manufacturers Association (NEMA) were encouraged by the Food and Drug Administration (FDA) to institute a standard format, spurred by the rapid growth of X-ray computed tomography usage in the 1970’s and the proliferation of incompatible manufacturer-specific image formats. The new standard provided for (1) for the transfer and display of digital images, (2) development of picture archiving and communication systems (PACS) with interfaces to other health care information systems, and (3) allowed for the creation of diagnostic databases. The DICOM standard has undergone numerous enhancements as manufacturers improved and developed new radiological equipment but retains backward compatibility. Unfortunately, DICOM has seen only limited adoption for small animal pre-clinical research. Pre-clinical modality manufacturers have either developed their own proprietary image formats or used a minimum subset of features within the clinical DICOM standard. Equipment intended for human imaging that is re-purposed for animal imaging generally produces DICOM, but typically suffers from a lack of animal-specific identifying and descriptive parameters.

With increased attention to issues of translation of research from bench to bedside, co-clinical (animal and human) trials, multiple-mouse acquisitions and analysis, complex animal models, and quantitative imaging, there are small animal-oriented tasks that need to be supported to measure, record and process various small animal data in minimally labor-intensive methods.

To address these issues, The National Cancer Institute (NCI) Division of Cancer Treatment and Diagnosis (DCTD) and the Center for Biomedical Informatics and Information Technology (CBIIT), formed a DICOM working-group in 2013, comprised of members from National Laboratories, the DICOM standard, academia, contract research organizations (CRO’s), and modality manufacturers, in order to extend DICOM to better support small animal imaging and to employ established semantically interoperable terminology.

The new DICOM working group (WG30) realized early on the importance of recording pertinent data about the research animals and their conditions. Since this type of information potentially influences the interpretation and quantification of images, it is referred to as Image Acquisition Context Structured Report (SR) [1]. It includes information about anesthesia, mouse model (i.e., NOD.*Cg-Prkdc^scid^Il2rg^tm1Wjl^*/SzJ, C57BL/6, etc.), cancer (cell or fragment) model, animal date of birth, date and site of cell/fragment implant, date of fragment excision, and facility-vivarium information, etc.) [2,3,4,5,6,7].

Personalized medicine, the ability to define a therapy to a specific patient has inaugurated co-clinical studies, where in vivo pre-clinical and early clinical studies are closely associated. As part of this endeavor, pre-clinical researchers instituted the mouse hospital to provide the testing of various therapies and monitor responses with statistically significant datasets. Since many animals are involved, it is efficient to image multiple mice at the same time, if the equipment allows for it. The implementation of multi-mouse imaging requires careful attention to the identification of groups of animals and individual animals, whether using manual or automated recording methods. For interpretation and analysis, when a cohort of more than one mouse has been imaged in the multi-mouse field of view, it is desirable to split the multi-mouse images into single mouse images, with corresponding updated DICOM information in the derived image headers.

Presently, most pre-clinical researchers use either a spreadsheet or database for maintaining and tracking the animal model and the animal handling processes. This brief article is to provide co-clinical and pre-clinical researchers the method to convert their tracking spreadsheet into the small animal acquisition context structured report and linked to the DICOM image. To assist researchers to convert their spreadsheets into the respective structured report, we provide all the necessary files and toolkits on The Cancer Imaging Archive (TCIA) (University of Arkansas for Medical Sciences, Little Rock, AR, USA) website [8]. In addition to the required files, we also provide a pre-clinical example including a tracking spreadsheet used in a patient-derived xenograft study, associated MRI data, and the resulting image acquisition context SR.

To provide the researcher the ability to query between pre-clinical and clinical datasets, a one-to-one query, it became necessary to split the pre-clinical multi-rodent datasets into single images and linked to the appropriate acquisition context structured report. The process and algorithms to split any DICOM dataset are also provided and as an example, MRI data from a patient-derived xenograft study, is provided and uploaded to the TCIA website.

## 2. Methodology

### 2.1. Image Acquisition Context Structured Report

Requiring entry of all pre-clinical acquisition context parameters during an imaging session to populate image headers would be labor-intensive for the operator and burdensome to the modality vendor. Therefore, WG30 defined a separate object to encode the image acquisition context, and store and transmit it separately from the image objects. The approach selected was to encode the acquisition context information using structured terminology in trees and lists of coded name-value pairs, preferably using well-established coded concepts that were already defined by other organizations such as the NCI Metathesaurus [9], National Library of Medicine (NLM) Unified Medical Language System (UMLS) [10], the Mouse Genome Informatics [11], other DICOM working groups (including WG25 on Veterinary Medicine), as well as other standards (American Society of Anesthesiologists [12], and the Systemized Nomenclature of Medicine Clinical Terms (SNOMED CT)) [13]. The DICOM Structured Report (SR) object [14] was selected to encode the separate Acquisition Context information. The specifications of the DICOM SR template and corresponding codes for small animal imaging was finalized and accepted in 2016 by the DICOM Standards Committee as Supplement 187 [15] and has been available in the current release of the complete standard since that time [16].

In the case of the patient-derived models that are the subject of this report, the experimental animals that were imaged are tracked in a spreadsheet that maps each animal to the NCI Patient-Derived-Models Repository (PDMR) database [2] using specified identifiers, and records in various columns descriptive information extracted from the PDMR database as well as image acquisition context information. We developed a procedure to:

- extract the worksheet from the spreadsheet in tab-delimited text form (tabdelimitedtoxml),
- convert each row to Extensible Markup Language (XML) files, one for each subject, using an eXtensible Stylesheet Language (XSL-T),
- convert each of the XML files into an XML-representation of a DICOM Structured Report conforming to the Pre-clinical Small Animal Image Acquisition Context SR Information Object Definition (IOD) (TID 8101), using an XSL-T stylesheet,
- use the XML to DICOM SR conversion capabilities of the PixelMed Java DICOM toolkit [17] to create DICOM SR files,
- upload DICOM SR files to TCIA for curation and inclusion in their archive alongside the split images.

XSL-T was chosen as the language in which to implement this process, since the transformations can be described in a declarative manner as a tree rewriting process, and XSL-T supports the transformation of textual information in other forms using string processing functions. The source code (individualxmltodicomsrxml) for the conversion process is available in a Supplementary File that accompanies this paper. An equally valid alternative would have been to read the tab-delimited text data into procedural code and construct the binary DICOM SR name-value pair trees using an SR-aware toolkit API (which the PixelMed toolkit also provides), but the XSL-T seemed well suited to this application.

The resulting DICOM SR files were validated for basic DICOM compliance using the dciodvfy tool [18], and the compliance with TID 8101 was tested using the DicomSRValidator tool [19].

This initial implementation of the DICOM SR TID 8101 Acquisition Context files encoded parameters of the exogenous graft (fragment origin, implant location, and date of implant and excision), for example (with the binary representation formatted in the style of [14]), which illustrates the name-value pairs containing the exogenous graft information encoded using standard terminology sources as specified by the DICOM template:

CONTAINER: (127001,DCM,“Preclinical Small Animal Imaging Acquisition Context”) [SEPARATE] (DCMR,8101)
  HAS CONCEPT MOD: CODE: (121049,DCM,“Language of Content Item and Descendants”) = (eng,RFC5646,“English”)
    HAS CONCEPT MOD: CODE: (121046,DCM,“Country of Language”) = (US,ISO3166_1,“United States”)
  HAS OBS CONTEXT: PNAME: (121008,DCM,“Person Observer Name”) = “SAIP^Imager”
  CONTAINS: CONTAINER: (127400,DCM,“Exogenous substance”) [SEPARATE]
    CONTAINS: CODE: (127460,DCM,“Tumor Graft”) = (2092003,SCT,“Melanoma”)
      HAS PROPERTIES: DATETIME: (111526,DCM,“DateTime Started”) = “20190722”
      HAS PROPERTIES: DATETIME: (111527,DCM,“DateTime Ended”) = “20190904”
      HAS PROPERTIES: TEXT: (111529,DCM,“Brand Name”) = “425362-245-T”
      HAS PROPERTIES: CODE: (410675002,SCT,“Route of administration”) = (34206005,SCT,“Subcutaneous route”)
      HAS PROPERTIES: CODE: (272737002,SCT,“Site of”) = (58602004,SCT,“Flank”)
        HAS CONCEPT MOD: CODE: (272741003,SCT,“Laterality”) = (24028007,SCT,“Right”)
      HAS PROPERTIES: CODE: (127401,DCM,“Tissue of origin”) = (39937001,SCT,“Skin”)
    HAS PROPERTIES: CODE: (127402,DCM,“Taxonomic rank of origin”) = (337915000,SCT,“Homo sapiens”)

Note the extensive use of SNOMED CT codes, and the use of DICOM-defined (DCM) codes when no appropriate SNOMED CT codes were available.

DICOM had previously added support for the species (taxon) and breed of animals for veterinary applications, but a description of the animal strain was also desirable. WG30 added this information in a DICOM Change Proposal (CP) 1478 [20]. Since neither the species nor strain could be entered at the modality acquisition console, nor the anatomical orientation type, these were added during the TCIA curation process. For example:

Anatomical Orientation Type (0010,2210) = “QUADRUPED”
Patient Species Description (0010,2201) = “Mus musculus”
Patient Species Code Sequence (0010,2202):
  > Code Value = “447612001”
  > Coding Scheme Designator = “SCT”
  > Code Meaning = “Mus musculus”
Strain Description (0010,0212) = “NOD.Cg-Prkdc<scid> Il2rg<tm1Wjl>/SzJ”
Strain Nomenclature (0010,0213) = “MGI_2013”
Strain Code Sequence (0010,0219):
  > Code Value = “3577020”
  > Coding Scheme Designator = “MGI”
  > Code Meaning = “NOD.Cg-Prkdc<scid> Il2rg<tm1Wjl>/SzJ”

### 2.2. Splitting and Identification of Multi-Mouse Images

In normal human clinical imaging, a single subject is imaged (the patient), and various identifying and descriptive attributes are encoded. The same attributes are populated when a single mouse is imaged, or when a single mouse image is split (derived from) a multi-mouse image. When multiple animals are imaged simultaneously, it is conventional to populate the single Patient’s Name and Patient’s ID attributes with values that indicate the combination of mice, using some local site-specific syntactic convention, which includes a lexical means of identifying the relative positions of the animals within the bore.

Recognizing the weakness of this approach, WG30 formalized encoding the identification of multiple subjects within one image, as well as a reference mechanism to encode which of such a group of subjects a single mouse image might have been derived from. This information was standardized in DICOM CP 1457 [21] to form the Patient Group Macro [22]. However, in practice, neither dedicated small animal nor re-purposed human imaging equipment is capable of populating these yet, so the historical approach of overloading the Patient’s Name and Patient’s ID attributes remains necessary.

Accordingly, we adopted a procedure in which any multi-mouse DICOM images can be split into equal sized volumes implementing a Python script (Python Software Foundation, Beaverton, OR, USA) based on the Pydicom library [23]. The multi-mouse image splitting source code is a single Python file (pydicom_split.py) and available at the GitHub repository [24].

This Python script implements the affine transform to split the multi-mouse DICOM images into a user-specified number of equally sized volumes along the radiological orientation (trans-axial, coronal, sagittal) without changing the pixel spacing values. Multi-mouse DICOM images contains more than one subject/patient sharing the same set of Universal Unique Identifiers (UUIDs). Therefore, it was necessary to encode a random UUID to avoid UUID conflicts. Implementing the Python script uuid4() [25] updates the UUID with a random UUID which is then encoded as an Object ID (OID) by adding “2.25.” in front of the decimal encoding of the random UUID [26]. It was also determined that a link to the original multi-mouse image was necessary and two DICOM attributes DerivativeDescription (0008,2111) and DerivationImageSequence (0008,9124) were added as well as updates to the Patient’s (subject) Name (0010,0010) and Patient’s ID (0010,0020) for identifying each individual subject. The original subject/patient name and ID are saved in the DICOM attribute Source Patient Group Identification Sequence (0010,0026) as SQ (sequence) data elements for reference to the split images. It should be noted that the naming schema implemented in the script is based on the imaging sites established naming convention and should be modified for images acquired at other sites, a summary of the modified DICOM attributes is provided in Table 1.

**Figure 1:**
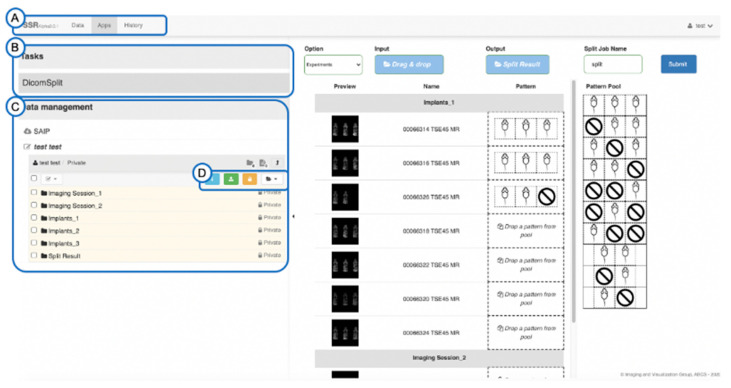
Graphic User Interface (GUI) for batch processing of multi-mouse DICOM images. (**A**) The Navigation Tab for Data Management, Tasks (Workflows) Execution, and Record Checking, (**B**) Available Tasks (DicomSplit task is shown as an example), (**C**) The Data Management Panel for data selection (DICOM PACS server or workstation), (**D**) Data Manipulation Actions provided by Girder [27] for image upload, download, and folder creation.

**Figure 2:**
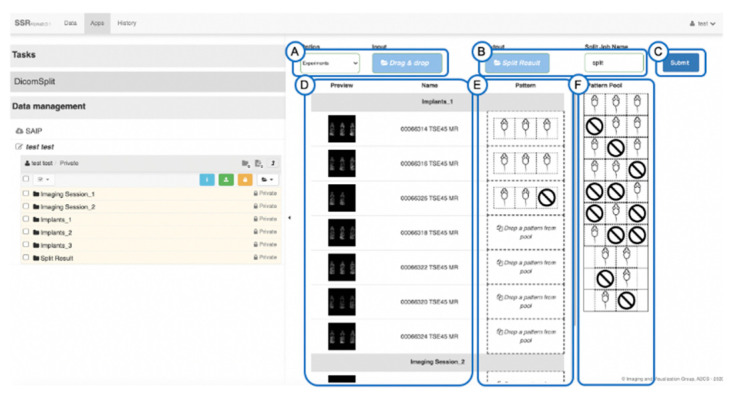
DICOM Split Task GUI. (**A**) Mode selection: allows the user to choose data root folder, either multiple or a single image, (**B**) select Output location, (**C**) Execute the split task in the background, (**D**) Overview panel displays image thumbnails of user-selected datasets. (**E**) Split Patterns selected by users for each dataset. (**F**). The Split Pattern for predefined mice arrangements. The predefined split patterns are drag-dropped from panel (**F**) to (**E**).

The Python script can be launched from the command line with manually defined parameters specifying mice arrangements, and all parameters are documented in the source code. Since pre-clinical imaging sites perform numerous multi-mouse datasets, the Python script was integrated in a web-based batch processing workflow [27] and based on the Girder data management platform, (Kitware Inc., Clifton Park, New York, NY, USA), an open-source software [28]. Figure 1 and Figure 2 provide a summary of the batch processing workflow.

The web-based User Interface (UI) allows the operator to visually review the arrangement of mice within the selected image datasets, select predefined splitting patterns, and perform the DICOM split in a batch mode. At the conclusion of the Python script, the split images can be reviewed, and the split image datasets downloaded as a zip file.

## 3. Results

The small animal acquisition context structured report (TID 8101) was initially implemented using MRI datasets of a patient-derived xenograft mouse model of urothelial bladder cancer in a drug challenge study [29,30] and uploaded to The Cancer Imaging Archive (TCIA). This enhancement allows co-clinical researchers the ability to query a disease, for example in TCIA, and obtain both clinical and pre-clinical datasets. To assist pre-clinical and co-clinical researchers to implement TID 8101, the MRI dataset and all associated files (spreadsheet for tracking the mice and all associated programs for conversion of tracking spreadsheets or databases to the associated image acquisition context spreadsheet) were uploaded to The Cancer Imaging Archive (TCIA) [8,29,30] for public dissemination. Furthermore, since queries require a one-to-one relation and the modality vendors at the present time are unable to provide the mouse location within the modality field-of-view, it was necessary to split multi-mouse DICOM images into their respective single mouse DICOM images with links to the corresponding acquisition context report. The DICOM multi-mouse splitting Python script and the Girder data management platform for implementing a web-based batch splitting routine are accessible from their respective websites [24,27]. For those researchers interested in testing their multi-modality splitting algorithms, multi-mouse and their corresponding single split MRI datasets (as an example) with the encoded small animal acquisition parameters, are provided on The Cancer Image Archive (TCIA) website.

## 4. Discussion

The small animal imaging community and the National Cancer Institute realized that to advance co-clinical research and to address the issues of translation from bench to bedside, the DICOM information that was developed for clinical images needed to be extended to pre-clinical data. The interpretation of pre-clinical data is influenced by numerous factors, such as anesthesia, mouse model (genetically engineered, mouse strain), cancer model (cell or fragment, orthotopic, xenograft), animal date of birth, date and site of cell/fragment implant, date of fragment excision, and facility-vivarium information, which are necessary to include in the pre-clinical DICOM. The DICOM WG30 group noted that the addition of the small animal data to the radiological image header should be performed in a minimally labor-intensive process, the Acquisition Context Structure Report template was utilized and developed the Pre-clinical Small Animal Image Acquisition Context (TID 8101). Additionally, to provide researchers the ability to cross-reference human-to-mouse anatomies, the mapping and harmonization developed by the Mouse-Human Anatomy Project was implemented.

Pre-clinical researchers implement mouse hotels to improve experimental statistics and to increase the number of mice within a cohort and the number of cohorts (vehicle, single, and combination drugs). Unfortunately, it is impossible to query a single mouse within the multi-mouse image with a single (human) radiological data. Therefore, a multi-mouse image splitting routine was implemented with unique UID’s with the ability to cross-reference to the original multi-mouse image.

In conclusion, this pre-clinical enhancement to DICOM enhances the researcher’s ability to query pre-clinical and clinical datasets using standard vocabularies and enhance co-clinical studies.

## Figures and Tables

**Table 1:** Modified Digital Imaging and Communications in Medicine (DICOM) Attributes for Multi-Mouse Image Split.

DICOM Tag	DICOM Attribute	Action
(0008,0018)	SOPInstanceUID	Create new “2.25.” decimal encoded OID per X.667
(0008,2111)	DerivationDescription	Added string attribute for reference purpose
(0008,9124)	DerivationImageSequence	Added SQ attribute for referencing back to original SOPClassUID and SOPInstanceUID
(0010,0010)	PatientName	Create new patient name based on the original patient name
(0010,0020)	PatientID	Create new patient ID based on the original patient ID
(0010,0026)	SourcePatientGroupIdentificationSequence	Added attribute stores the original patient name and ID
(0020,000d)	StudyInstanceUID	Create new “2.25.” decimal encoded OID per X.667
(0020,000e)	SeriesInstanceUID	Create new “2.25.” decimal encoded ODI per X.667
(0020,0011)	SeriesNumber	Update series number based on the original series number
(0088,0140)	StorageMediaFileSetUID	Create new “2.25.” decimal encoded OID per X.667

## Data Availability

Tatum JL, Kalen JD, Jacobs PM, Ileva LV, Riffle LA, Keita S, Patel N, Sanders C, James A, Difilippantonio S, Thang L, Hollingshead MG, Phillips J, Edmondson E, Evrard Y, Clunie DA, Liu Y, Smith KE, Wagner U, Freymann JB, Kirby J, Doroshow JH. (2020). Imaging *characterization of a metastatic patient derived model of melanoma: PDMR-425362-245-T* [Data set]. The Cancer Imaging Archive. https://doi.org/10.7937/TCIA.2020.7YRS-7J97.

## Supplementary Materials

The following are available online at https://www.mdpi.com/2379-1381/7/1/1/s1, The source code (individualxmltodicomsrxml) for the conversion process.

## Abbreviations

DICOM	Digital Imaging and Communications in Medicine
MRI	Magnetic Resonance Imaging
TCIA	The Cancer Imaging Archive
